# Prognostic significance of positive margin-informed tumor status in esophageal squamous cell carcinoma using next-generation sequencing

**DOI:** 10.1093/oncolo/oyag337

**Published:** 2026-08-22

**Authors:** Di Lu, Yu Tong, Chunhong He, Zhiming Chen, Zhizhi Wang, Jingwen Liu, Jiani C Yin, Shaobin Li, Shijie Mai, Xuguang Rao, Kaican Cai

**Affiliations:** Department of Thoracic Surgery, Nanfang Hospital, Southern Medical University, Guangzhou, Guangdong 510515, China; Department of Thoracic Surgery, Nanfang Hospital, Southern Medical University, Guangzhou, Guangdong 510515, China; Geneseeq Research Institute, Nanjing Geneseeq Technology Inc., Nanjing, Jiangsu 210032, China; Department of Thoracic Surgery, Nanfang Hospital, Southern Medical University, Guangzhou, Guangdong 510515, China; Department of Thoracic Surgery, Nanfang Hospital, Southern Medical University, Guangzhou, Guangdong 510515, China; Geneseeq Research Institute, Nanjing Geneseeq Technology Inc., Nanjing, Jiangsu 210032, China; Geneseeq Research Institute, Nanjing Geneseeq Technology Inc., Nanjing, Jiangsu 210032, China; Department of Thoracic Surgery, Nanfang Hospital, Southern Medical University, Guangzhou, Guangdong 510515, China; Department of Thoracic Surgery, Nanfang Hospital, Southern Medical University, Guangzhou, Guangdong 510515, China; Department of Thoracic Surgery, Nanfang Hospital, Southern Medical University, Guangzhou, Guangdong 510515, China; Department of Thoracic Surgery, Nanfang Hospital, Southern Medical University, Guangzhou, Guangdong 510515, China

**Keywords:** esophageal squamous cell carcinoma, margin-informed tumor, minimal residual disease, *TP53*, gene set enrichment analysis, immune cell infiltration

## Abstract

**Background:**

Reliable prognostic biomarkers are urgently needed for operable esophageal squamous cell carcinoma (ESCC), as conventional histopathology often fails to detect minimal residual disease and accurately predict patient outcomes.

**Patients and Methods:**

This study investigated the mutational patterns in ESCC and the prognostic value of shared mutations between tumors and margins (margin-informed tumor, MiT) using next-generation sequencing. We analyzed tumor, margin, and normal tissues from a total of 70 patients with operable ESCC, including 48 from a retrospective cohort and 22 from a prospective cohort.

**Results:**

*TP53* mutations were more prevalent in tumors than in margins or normal tissues (retrospective, *P* <.0001; prospective, *P* = .02), while *NOTCH1* mutations were more frequent in normal or margin tissues (retrospective, *P* = .0002; prospective, *P* = .07). Positive MiT was observed in 16.7% of the retrospective cohort and 45.5% of the prospective cohort, respectively, with all patients exhibiting over 50% shared mutations experiencing disease progression. Patients with identical *TP53* mutations in both tumor and margin tissues (*TP53*-MiT) exhibited significantly worse survival (retrospective, hazard ratio [HR] = 3.32, 95% confidence interval [CI], 1.11-9.91, *P* = .03; prospective, HR = 4.35, 95% CI, 0.87-21.63, *P* = .05). *TP53* mutations were associated with tumorigenic pathways and low immune infiltration fraction.

**Conclusion:**

Our findings underscore the clinical importance of MiT, particularly *TP53*-MiT, in postoperative prognosis. Integrating *TP53*-MiT assessment into postoperative evaluations could refine risk stratification and guide adjuvant management better.

## Graphical abstract

**Graphical Abstract:**
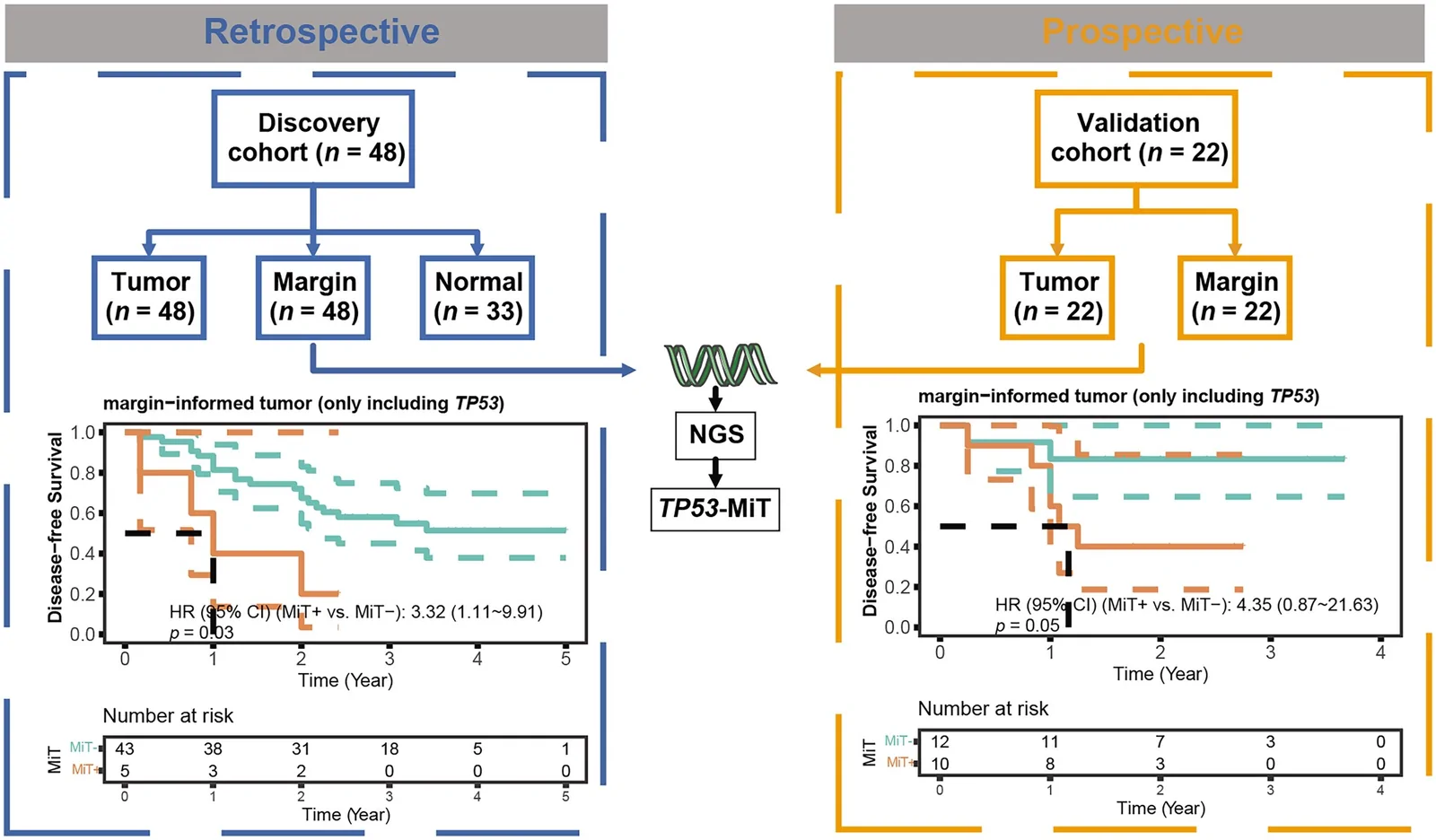

Implications for Practice Current histopathological assessment of surgical margins in esophageal squamous cell carcinoma (ESCC) often misses minimal residual disease. This study demonstrates that next-generation sequencing (NGS) can detect tumor-specific *TP53* mutations in histologically tumor-free margins. The *TP53*-MiT status is a robust predictor of disease recurrence and poor survival. For clinicians, this highlights a critical subgroup of patients who, despite successful surgery, remain at high risk. Adopting molecular margin assessment could transform postoperative care by identifying patients who require intensified adjuvant therapy or rigorous surveillance, thereby advancing personalized treatment strategies in ESCC.

## Introduction

Esophageal squamous cell carcinoma (ESCC) is the fourth most prevalent cancer in China and remains a major cause of cancer-related mortality.^1^ Surgical treatment is recommended for patients with operable ESCC.^2^^,^^3^ However, postoperative outcomes vary considerably, largely due to prognostic factors such as surgical margin status.^4–6^ Emerging evidence has shown that the existence of gene alterations in surgical margins increases the likelihood of tumor recurrence and necessitates secondary treatments such as adjuvant or salvage radiation.^7–9^ Patients with positive surgical margins have a markedly lower 5-year survival rate, and adjuvant therapy offers limited improvement.^10^ Although conventional histopathological examination remains the gold standard for intraoperative margin assessment, its postoperative prognostic value is limited by tissue heterogeneity, sampling errors, and inconsistent assessment criteria.^4^ Disease recurrence can occur even after histologically confirmed negative margins.

Next-generation sequencing (NGS) provides high-resolution molecular insights into tumor biology,^11^^,^^12^ and can detect minimal residual disease (MRD) missed by conventional pathology. Previous studies have demonstrated that positive postsurgical circulating tumor DNA (ctDNA) in patients with esophageal adenocarcinoma who underwent R0 resection correlates with worse disease-free survival (DFS).^13^ This underscores the potential of liquid biopsies in identifying patients at higher risk of relapse. Similarly, tumor mutations detected in resected bronchial margins using NGS have been associated with early relapse in patients with non-small cell lung cancer.^14^ Building on these insights, NGS could be particularly promising for examining surgical margins in ESCC patients, potentially improving the detection of MRD, thereby enhancing prognostication and informing postoperative management.

Recent studies have shown that histologically normal esophageal tissues can harbor mutations in key genes, such as *TP53* and *NOTCH1*.^15^ However, comparative analyses of mutation landscapes across different tissue types, particularly tumor, margin, and normal tissues, remain inadequately explored. To fill this knowledge gap, we conducted a comprehensive analysis of the mutational profiles of paired tumor, margin, and normal esophageal tissues from patients with resectable ESCC using NGS. Focusing on the key genes *TP53* and *NOTCH1*, we aimed to elucidate their roles across different tissue types. We introduce the concept of the “margin-informed tumor” (MiT) status, defined by shared mutations between tumor and margin tissues, as a novel MRD indicator. We hypothesize that the MiT status could serve as a prognostic marker for DFS, offering valuable insights for postoperative decision-making beyond traditional histopathological assessments.

## Patients and methods

### Patients and sample collection

This single-center study includes a total of 70 patients diagnosed with operable ESCC who underwent surgery at the Department of Thoracic Surgery, Nanfang Hospital, Southern Medical University, between 2019 and 2022, adhering to the STROBE reporting criteria.^16^ All cases were confirmed by cytopathological and histological examination. Patients who had received neoadjuvant therapy or had other conditions, such as leukemia, neurodegenerative diseases, or other tumors, were excluded. Tissue samples, including esophageal tumors, surgical margins, and normal tissues, were collected. Tumor samples were required to have a tumor cell content of at least 20%. Surgical margins were defined as tissue specimens obtained from the proximal and/or distal transection margins (the actual cut ends) of the resected esophagus, which were histologically confirmed to be free of tumor cells via pathological examination before molecular analysis. Normal tissues were defined as non-tumorous tissues taken at the distal part of the esophagus, farthest from the tumor site. In addition to tissue samples, matched peripheral blood (10 mL) was collected from each patient in EDTA tubes prior to the surgery. White blood cells (WBCs) were isolated from peripheral blood and stored at −80 °C until DNA extraction for use as matched germline controls. The retrospective cohort involves a total of 48 patients. Of these, 33 patients had qualified paired tumor, margin, and normal tissue samples, while 15 patients provided only paired tumor and margin samples without normal tissues.

To further validate our findings in the retrospective cohort, we accrued an additional 22 prospective patients adhering to the same inclusion and exclusion criteria during the period from 2020 to 2023. Tissue samples, including esophageal tumors and surgical margins, were collected. All 22 patients had qualified paired tumor and margin samples.

All collected samples were subjected to NGS using a pan-cancer panel of 425 genes (GeneseeqPrime^TM^) at a College of American Pathologists (CAP)-accredited, Clinical Laboratory Improvement Amendments (CLIA)-certified, and International Organization for Standardization (ISO) 15189-accredited laboratory (Nanjing Geneseeq Technology Inc., Nanjing, China). This study was approved by the Research Ethics Board of the Nanfang Hospital (No. NFEC-2020-069, approval: 21/4/2020) and was conducted in accordance with the Declaration of Helsinki. Written informed consent was obtained from all the participants.

### DNA extraction, quantification, and library preparation

Formalin-fixed paraffin-embedded (FFPE) samples from surgical sections were used for genomic profiling. Genomic DNA was extracted using the QIAamp DNA FFPE Tissue Kit (Qiagen, Hilden, Germany) and the QIAamp DNA Blood Mini Kit (Qiagen, Hilden, Germany), qualified by Nanodrop 2000 (Thermo Fisher Scientific, Waltham, MA, USA), and quantified with a dsDNA HS assay kit on a Qubit 3.0 fluorometer (Life Technology, Carlsbad, CA, USA). Subsequent library preparation was carried out using the KAPA Hyper Prep Kit (KAPA Biosystems, Wilmington, MA, USA) as previously described.^17^^,^^18^ Briefly, fragmented genomic DNA underwent end-repairing, A-tailing, indexing with adapters, and magnetic bead DNA purification (Beckman Coulter, Mississauga, Canada). Target enrichment was performed using customized xGen lockdown probes (Integrated DNA Technologies, Coralville, IA, USA) targeting 425 cancer-related genes (Nanjing Geneseeq Technology Inc., Nanjing, China). Sequencing was performed on the Illumina HiSeq4000 platform followed by data analysis.

### Mutation calling

The following steps were employed to obtain qualified FASTQ files from the Illumina output: (i) removal of *N* bases with a quality score below 15 using Trimmomatic^19^ (https://github.com/usadellab/Trimmomatic) and (ii) elimination of PCR duplicates via Picard (https://broadinstitute.github.io/picard/). The resulting clean paired-end reads were aligned to the human genome reference (GRCh37, hg19) using the Burrows–Wheeler Aligner (https://github.com/lh3/bwa/tree/master/bwakit) and realigned around indels using GATK4 (https://github.com/broadinstitute/gatk/releases). Single nucleotide variants (SNVs) and indels were identified via VarScan2^20^ (https://varscan.sourceforge.net/) and annotated by ANNOVAR (https://annovar.openbioinformatics.org/en/latest/). SNVs/indels were defined using a variant allele frequency (VAF) threshold of 0.01 and a *P* threshold of .05. Given the differences in tumor purity between tumor tissues and margin samples, additional filters were applied for SNVs/indels from different tissue sources: (i) read depth ≥ 30; (ii) unique variant supporting reads of indels ≥ 3 and VAF ≥ 0.5% for cancer-related hotspot SNVs and indels in tumor tissues; (iii) unique variant supporting reads of indels ≥ 2 and VAF ≥ 0.5% for cancer-related hotspot SNVs and indels in margin tissues; (iv) unique variant supporting reads of indels ≥ 5 and VAF ≥ 1% for nonhotspot SNVs and indels in tumor tissues; (v) unique variant supporting reads ≥ 4 and VAF ≥ 0.5% for nonhotspot SNVs and indels in margin tissues; (vi) supporting reads mapped to both directions of strands; and (vii) exclusion of SNVs with an occurrence frequency >1% in the 1000 Genomes Project or the ExAC database.^21^ Furthermore, matched WBC DNA was used as negative controls to filter germline and clonal hematopoiesis-associated variants.

### Identification of MiT status

A positive MiT status was defined as at least 1 identical mutation shared between paired tumor and margin samples, requiring the same chromosome, genomic position, reference/alternate alleles, and indel representation. Conversely, a negative MiT status indicated no shared mutation.

### Impact of clinical features on the MiT status

To determine whether the relationship between DFS and MiT status is influenced by other clinical factors, we incorporated multiple covariates in our analysis, including the use of adjuvant therapy (yes vs no), advanced clinical stage (III and IV vs I and II), lymph node metastasis (LNM, positive vs negative), vascular invasion (positive vs negative), nerve invasion (positive vs negative), tumor size (≥ 4 cm vs < 4 cm), and tumor location (middle third and middle-to-lower esophagus vs lower esophagus).

### External GSEA analysis and immune cell infiltration fraction calculation

To further explore the molecular pathways affected by genetic alterations in ESCC, we obtained and analyzed both RNA‐seq and whole-exome sequencing data from the TCGA esophageal carcinoma cohort (*n* = 94, https://portal.gdc.cancer.gov/). Patients were divided into subgroups based on the presence or absence of *TP53* and *NOTCH1* gene alterations, and differentially expressed genes (DEGs) were identified via DESeq2.^22^ Gene set enrichment analysis (GSEA)^23^ was performed on different gene sets from the Molecular Signatures Database (MSigDB) hosted by the Broad Institute to understand the molecular pathways affected by genetic alterations in ESCC. Enrichment scores, normalized enrichment scores (NES), and false discovery rates (FDR) were calculated to identify significant pathways. Tumor immune infiltration was estimated in different groups employing the CIBERSORT algorithm via the TIMER2.0 database (http://timer.cistrome.org/).

### Statistical analysis

Categorical variables were expressed as frequencies and percentages, and group comparisons were performed using Fisher’s exact test. Quantitative variables were presented as medians and interquartile ranges due to their skewed distributions. Comparisons of the number of mutations between two groups were conducted using the Wilcoxon rank-sum test, while comparisons among three groups were performed using the Kruskal–Wallis test. The Jonckheere-Terpstra test was applied to assess whether there was a statistically significant increasing or decreasing trend in gene mutation counts across different tissue types. Survival distributions of groups were described by Kaplan–Meier curves and compared by the log-rank test. For GSEA, nominal *P*-values were adjusted for multiple testing using the FDR method, and FDR-adjusted *P*-values were reported in the enrichment plots. A two-sided *P*-value or an FDR-adjusted *P*-value less than 0.05 (*P <.05, **P <.01, ***P <.001, ****P <.0001) was considered significant unless indicated otherwise. All statistical analyses were performed using R 4.3.3.

## Results

### Clinical and overall genetic characteristics of the study cohorts

**Figure 1.**
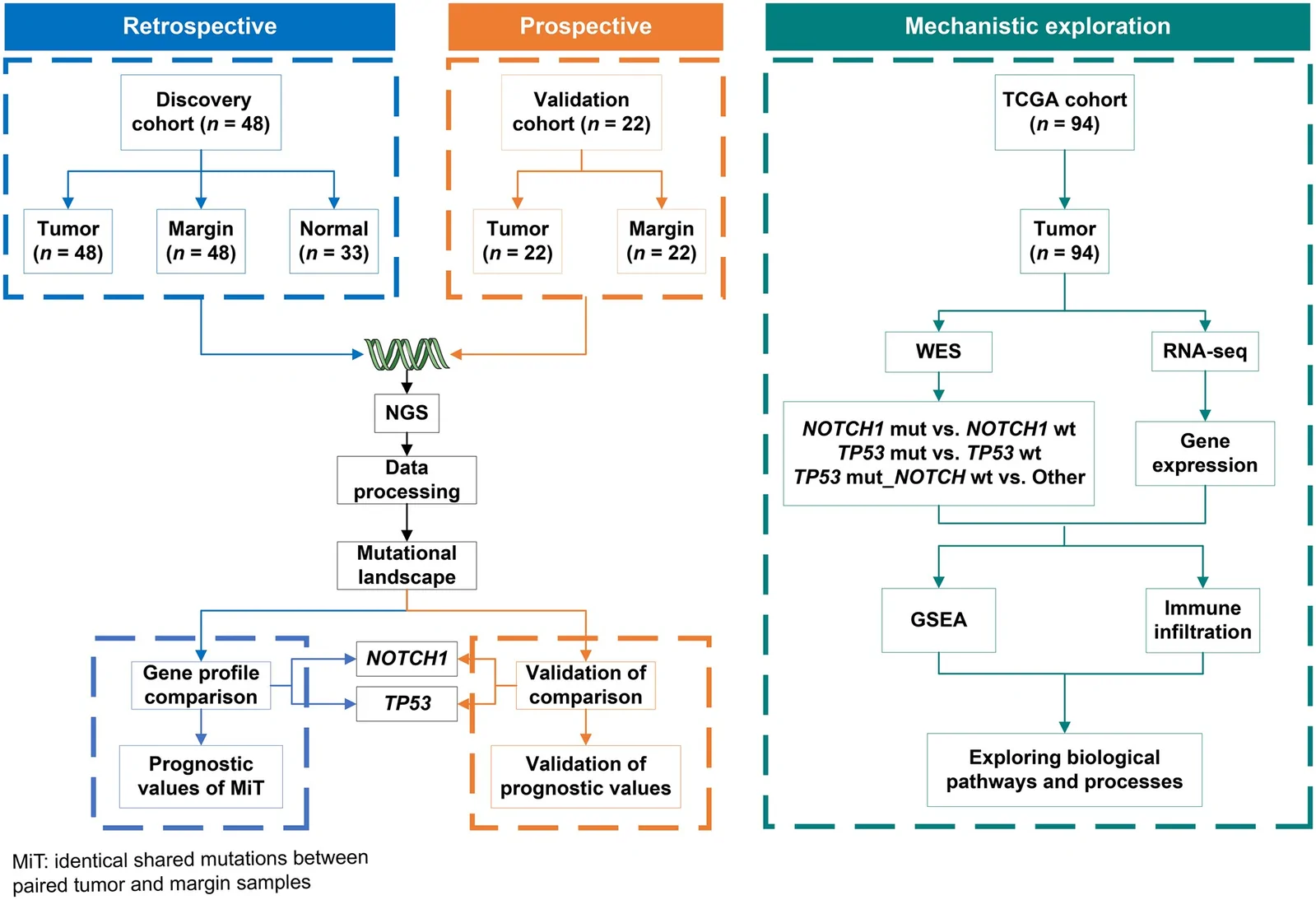
Schematic of the study design. The study encompassed a discovery cohort (*n* = 48) and a validation cohort (*n* = 22) to assess and validate gene profiles, mutational landscapes, and the prognostic significance of *TP53* and *NOTCH1* mutations in esophageal squamous cell carcinoma (ESCC). The mechanistic evaluations of *TP53* and *NOTCH1* in ESCC were investigated by an external TCGA cohort (*n* = 94). GSEA, Gene Set Enrichment Analysis; MiT, margin-informed tumor; mut, mutation; NGS, next-generation sequencing; RNA-seq, RNA sequencing; WES, whole-exome sequencing; wt, wild-type.

A comprehensive overview of our study design is depicted in Figure 1, and the clinicopathological characteristics of the cohorts are summarized in Table 1. In the retrospective cohort, 75.0% (36/48) of patients were male, with a median age of 63.0 years (interquartile range [IQR]: 55.8-67.2). Several cases demonstrated local tumor invasion, including vascular involvement in 6.3% and neural involvement in 4.2%. In addition, LNM was present in 14.6% of cases. Tumor locations varied, with 37.5% located in the middle third of the esophagus, 27.1% in the middle to lower, and 33.3% in the lower third. Notably, 62.5% of patients received adjuvant therapy, while none received neoadjuvant therapy. Regarding lifestyle factors, 43.8% of the patients were nonsmokers, and 70.8% reported no history of alcohol use (Table 1).

**Table 1.** Clinicopathological characteristics of patients in the retrospective and prospective cohorts.

Characteristics	Retrospective cohort (*n* = 48)	Prospective cohort (*n* = 22)
**Age**		
** Median (IQR)**	63.0 (55.8, 67.2)	59.5 (55.0, 65.8)
**Sex assigned at birth**		
** Female**	12 (25.0%)	7 (31.8%)
** Male**	36 (75.0%)	15 (68.2%)
**TNM stage**		
** I**	5 (10.4%)	1 (4.5%)
** II**	36 (75.0%)	9 (40.9%)
** III**	6 (12.5%)	9 (40.9%)
** IV**	1 (2.1%)	3 (13.6%)
**Pathological differentiation stage of tumors**		
** Well, well-moderately**	18 (37.5%)	5 (22.7%)
** Moderately, poorly**	25 (52.1%)	14 (63.6%)
** Poorly**	4 (8.3%)	2 (9.1%)
** Unknown**	1 (2.1%)	1 (4.5%)
**Tumor size**		
** Median (IQR)**	4.0 (3.0, 5.0)	4.1 (3.1, 4.6)
**Vascular invasion**		
** Positive**	3 (6.3%)	6 (27.3%)
** Negative**	44 (91.6%)	15 (68.2%)
** Unknown**	1 (2.1%)	1 (4.5%)
**Nerve invasion**		
** Positive**	2 (4.2%)	13 (59.1%)
** Negative**	44 (91.6%)	9 (40.9%)
** Unknown**	2 (4.2%)	0
**Lymph node metastasis**		
** Positive**	7 (14.6%)	11 (50.0%)
** Negative**	41 (85.4%)	11 (50.0%)
**Tumor site**		
** Middle third esophagus**	18 (37.5%)	7 (31.8%)
** Middle to lower esophagus**	13 (27.1%)	10 (45.5%)
** Lower third esophagus**	16 (33.3%)	5 (22.7%)
** Unknown**	1 (2.1%)	0
**Neoadjuvant therapy**		
** Yes**	0	0
** No**	48 (100%)	22 (100%)
**Adjuvant therapy**		
** Yes**	30 (62.5%)	9 (40.9%)
** No**	17 (35.4%)	13 (59.1%)
** Unknown**	1 (2.1%)	0
**Smoking history**		
** Never**	21 (43.8%)	10 (45.5%)
** Former**	4 (8.3%)	8 (36.4%)
** Current**	22 (45.8%)	4 (18.2%)
** Unknown**	1 (2.1%)	0
**Alcohol history**		
** Never**	34 (70.8%)	20 (91.0%)
** Former**	2 (4.2%)	0
** Current**	11 (22.9%)	1 (4.5%)
** Unknown**	1 (2.1%)	1 (4.5%)

Abbreviations: ESCC, esophageal squamous cell carcinoma; IQR, interquartile range.

In the prospective study cohort of 22 patients, 68.2% of patients were male, with a median age of 59.5 years (IQR: 55.0-65.8). Vascular invasion was observed in 27.3% of cases and neural invasion in 59.1%, while LNM was present in 50.0%. Tumor locations included the middle third of the esophagus in 31.8% of cases, the middle to lower in 45.5%, and the lower third in 22.7%. A notable 59.1% of patients did not undergo adjuvant therapy, and none received neoadjuvant therapy. In terms of lifestyle factors, 45.5% were nonsmokers, and 91.0% reported that they never drank alcohol (Table 1).

Genetic analysis of the retrospective cohort revealed a high prevalence of mutations detected in 95.8% (46 of 48) of tumor samples, with a median mutation count of 4 and an average of 5 mutations. Margin samples demonstrated mutations in 45.8% (22 of 48) of cases, with a median of 0 and an average of 2. In normal tissues, mutations were observed in 63.6% (21 of 33) of cases, with both the median and mean counts of 2 (Figure S1A).

### Shared mutational patterns between tumors and margins in the retrospective cohort

To gain deeper insights, we compared the mutational patterns between tumor and margin tissues. Identical mutations in paired margins and tumor samples were observed in 8 out of 48 (16.7%) patients, whereas no shared mutations were detected in other tissue pairings (Figure S1B). Among these 8 patients, 23 of 71 mutations (32.4%) were shared (Figure S1C). Key genes such as *TP53* (*n* = 6) and *NOTCH1* (*n* = 4) were responsible for many of these shared mutations (Figure S1D). The proportion of shared mutations varied among patients, ranging from 6.7% to 83.3% (Figure S1E). Interestingly, of the 2 patients with more than 50% shared mutations between tumor and margin tissues in the retrospective cohort (P02 and P04), both experienced disease progression (Figure S1F). However, no significant correlation was observed between the proportion of shared mutations and disease status (*P* = .88, Figure S1G).

### Distinct mutational landscapes of *NOTCH1* and *TP53* across tissue types in the retrospective cohort

**Figure 2.**
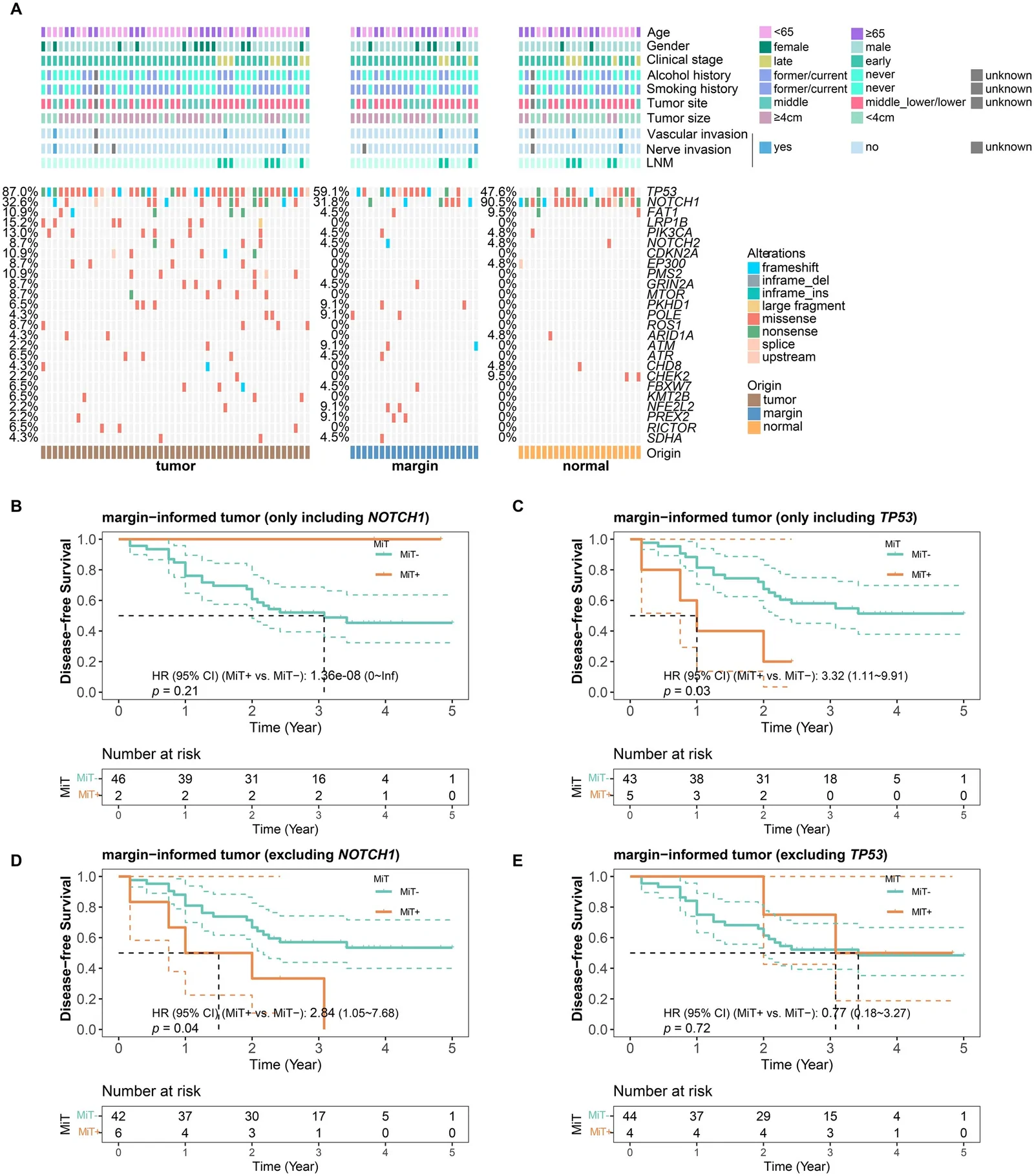
Mutation landscapes and prognostic significance of MiT status across tumor, margin, and normal tissues in the retrospective cohort. (A) Mutation profiles and clinical features of samples in the retrospective cohort. The mutation profiles and clinical features of 46 tumor samples, 22 margin samples and 21 normal samples with detectable mutations of the retrospective cohort were displayed in the left, middle, and right panels, respectively. Only the top 25 most frequently altered genes in the cohort were included. Each column represents an individual patient within the cohort. The upper sections illustrate clinicopathologic subtypes of patients, while the bottom sections depict different mutation types of genomic alterations. (B) Kaplan–Meier curves of DFS comparing *NOTCH1*-MiT positive and *NOTCH1*-MiT negative groups. (C) Kaplan–Meier curves of DFS comparing *TP53*-MiT positive and *TP53*-MiT negative groups. (D) Kaplan–Meier curves of DFS comparing non-*NOTCH1*-MiT positive and non-*NOTCH1*-MiT negative groups. (E) Kaplan–Meier curves of DFS comparing non-*TP53*-MiT positive and non-*TP53*-MiT negative groups. Differences in survival were evaluated using the log-rank test. HRs and 95% CI were calculated using Cox proportional hazards in survival analysis. Statistical significance is indicated as *P <.05, **P <.01, ***P <.001, ****P <.0001. del, deletion; DFS, disease-free survival; ESCC, esophageal squamous cell carcinoma; ins, insertion; MiT, margin-informed tumor.

Given that *TP53* and *NOTCH1* were identified as critical mutations shared between tumors and margins, and considering their established roles in the pathogenesis of ESCC, we focused subsequent analyses on these 2 genes. *NOTCH1* and *TP53* were the most frequently mutated genes in our study cohort, yet they displayed distinct mutation patterns across the three tissue types. To better characterize these patterns, we analyzed the mutation frequencies of *NOTCH1* and *TP53* in tumor, margin, and normal tissues. Specifically, *NOTCH1* mutations were observed in 32.6% of tumor samples, 31.8% of margin samples, and 90.5% of normal samples. *TP53* mutations were present in 87.0% of tumor samples, 59.1% of margin samples, and 47.6% of normal samples (Figure 2A). Interestingly, *NOTCH1* mutations were more frequent in normal tissues (57.6%) compared to tumor (31.3%) or margin (14.6%) samples (*P* = .0002). Conversely, the overall mutation rate of *TP53* was significantly higher in tumor (83.3%) than in margin (27.1%) or normal (30.3%) tissues (*P* <.0001, Figure S2A).

To further understand the mutation dynamics across the distinct tissue types, we examined the number of *NOTCH1* and *TP53* mutations in tumor, margin, and normal tissues, representing increasing distance from the tumor center. Our analysis revealed that *NOTCH1* did not exhibit a significant trend in mutation frequency across these tissue types (Jonckheere-Terpstra test, *P* = .15). In contrast, *TP53* showed a highly significant decline in mutations from tumor to margin to normal tissues (Jonckheere-Terpstra test, *P* <.0001, Figure S2B) in the retrospective cohort.

### Prognostic significance of MiT in the retrospective cohort

In light of the genetic overlap between the margin and tumor samples, we investigated the prognostic potential of margin-informed gene alterations with the concept of “MiT” (see Patients and Methods section), with a focus on *TP53* and *NOTCH1*. In the retrospective cohort analysis, incorporating solely *NOTCH1* mutations to define the MiT status (*NOTCH1*-MiT) did not yield significant prognostic differences (HR = 1.36e-08, 95% CI, 0-infinite, *P* = .21, Figure 2B). In contrast, patients with a positive *TP53*-MiT status exhibited worse DFS compared to those with negative *TP53*-MiT status (HR = 3.32, 95% CI, 1.11-9.91, *P* = .03, Figure 2C). Excluding *NOTCH1* mutations from the MiT assessment (non-*NOTCH1*-MiT) also indicated that patients with a negative MiT status had prolonged DFS (HR = 2.84, 95% CI, 1.05-7.68, *P* = .04, Figure 2D). Finally, excluding *TP53* mutations (non-*TP53*-MiT) did not reveal significant prognostic differences (HR = 0.77, 95% CI, 0.18-3.27, *P* = .72, Figure 2E).

### Validation using a prospective cohort of resectable ESCC

To validate our findings from the retrospective cohort, we conducted a comparative analysis of mutational patterns between tumor and margin tissues in our prospective cohort. Consistent with earlier findings, *NOTCH1* and *TP53* remained the most frequently altered genes. *TP53* mutations occurred in 100% of tumor tissues and 73.9% of margin tissues, while *NOTCH1* mutations were present in 30.4% of tumor tissues and 56.5% of margin tissues (Figure S3A). The prospective cohort demonstrated a higher mutation burden compared to the retrospective cohort, with tumor samples showing a median and average of 7 mutations and margin samples showing a median of 7 and an average of 6 mutations (Figure S3B). In the prospective cohort, 45.5% (10 of 22) patients harbored shared mutations in paired tumor and margin tissues (Figure S3C). Out of 117 mutations identified in tumor and margin tissues of these 10 patients, 34 (29.1%) were shared (Figure S3D). *TP53* (*n* = 10) and *NOTCH1* (*n* = 4) mutations were prominently featured among these shared mutations (Figure S3E). The distribution of shared mutations varied across patients, ranging from 4.0% to 85.7% (Figure S3F). Notably, all 3 patients with more than 50% shared mutations (P11, P14, and P17) exhibited disease progression (Figure S3G), consistent with our findings in the retrospective cohort. Nevertheless, when stratifying patients by disease progression status, no significant difference in the proportions of shared mutations was observed (*P* = .91, Figure S3H).

Consistent with the retrospective cohort, *NOTCH1* mutations were more prevalent in margin tissues, although this did not reach statistical significance (*P* = .07). On the other hand, *TP53* mutations were significantly more common in tumor tissues (*P* = .02, Figure S2C). Furthermore, *NOTCH1* displayed a significant increase in mutation frequency from tumor to margin tissues (Jonckheere-Terpstra test, *P* = .01). In contrast, *TP53* did not exhibit a significant mutation trend across tissues (Jonckheere-Terpstra test, *P* = .16, Figure S2D).

**Figure 3.**
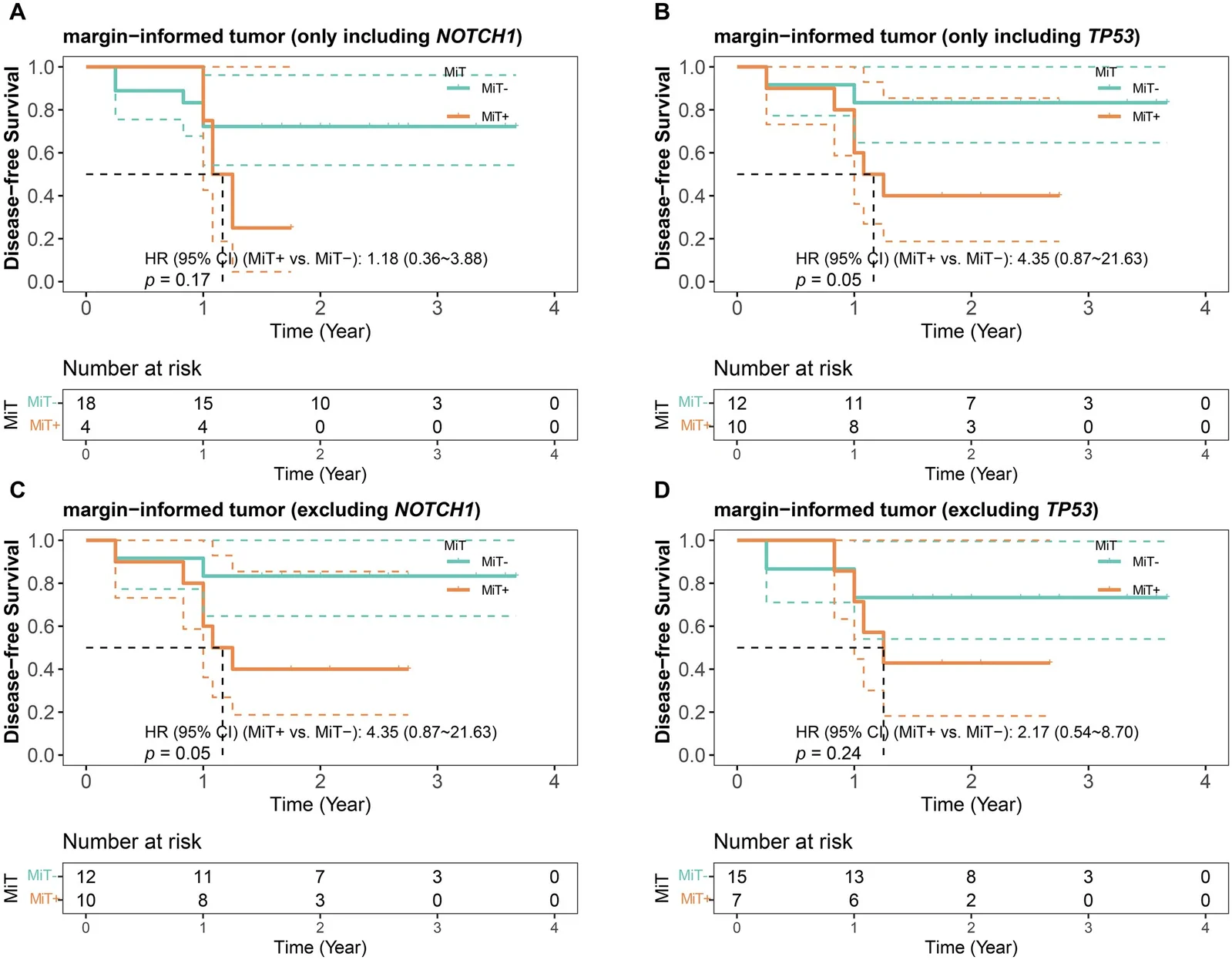
Validation of MiT status associations with DFS in the prospective cohort. (A) Kaplan–Meier curves of DFS comparing *NOTCH1*-MiT positive and *NOTCH1*-MiT negative groups. (B) Kaplan–Meier curves of DFS comparing *TP53*-MiT positive and *TP53*-MiT negative groups. (C) Kaplan–Meier curves of DFS comparing non-*NOTCH1*-MiT positive and non-*NOTCH1*-MiT negative groups. (D) Kaplan–Meier curves of DFS comparing non-*TP53*-MiT positive and non-*TP53*-MiT negative groups. Differences in survival were evaluated using the log-rank test. HRs and 95% CI were calculated using Cox proportional hazards in survival analysis. Statistical significance is indicated as * *P* <.05. DFS, disease-free survival; MiT, margin-informed tumor.

We also assessed the prognostic significance of MiT in the prospective cohort. Similar to the retrospective cohort, *NOTCH1*-MiT status did not have significant prognostic value for differentiating DFS (HR = 1.18, 95% CI, 0.36-3.88, *P* = .17, Figure 3A). Patients with a positive *TP53*-MiT status had significantly poorer DFS (HR = 4.35, 95% CI, 0.87-21.63, *P* = .05, Figure 3B), and non-*NOTCH1*-MiT status continued to identify patients with shorter DFS (HR = 4.35, 95% CI, 0.87-21.63, *P* = .05, Figure 3C). Conversely, non-*TP53*-MiT status did not significantly correlate with DFS (HR = 2.17, 95% CI, 0.54-8.70, *P* = .24, Figure 3D). In addition, margin mutation status alone did not significantly predict DFS in either the retrospective cohort (*P* = .55, Figure S4A) or the prospective cohort (*P* = .08, Figure S4B). Additionally, clinical features did not influence different MiT statuses in both cohorts (all *P* > .05, Figure S4C-F). Overall, these results corroborate the distinct mutational patterns observed in the retrospective cohort.

### *TP53* is more closely associated with ESCC tumorigenesis than *NOTCH1*

**Figure 4.**
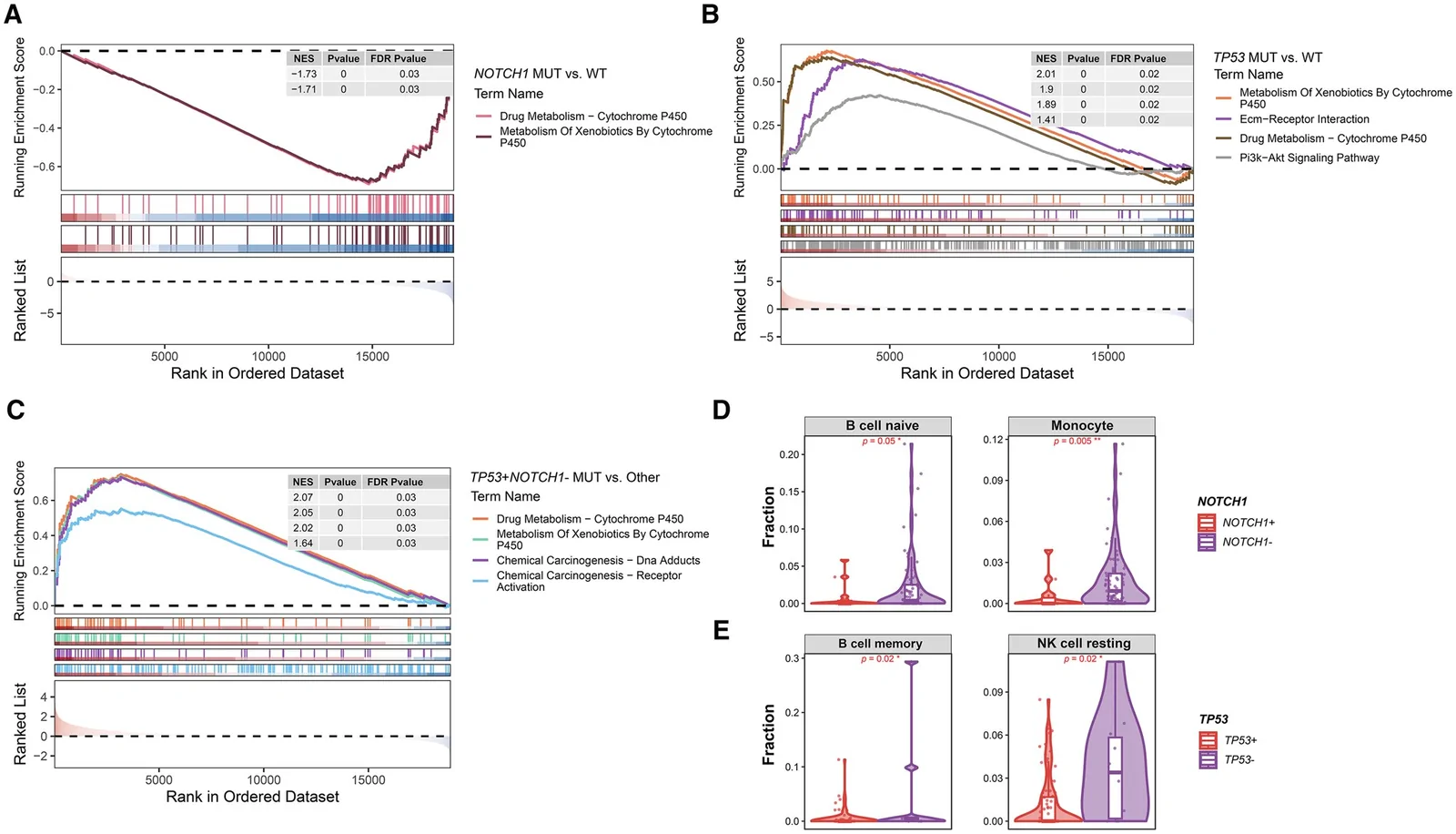
GSEA and immune infiltration analysis of the TCGA cohort. (A) GSEA enrichment plot for key MSigDB pathways comparing *NOTCH1* altered and *NOTCH1* wild-type groups. (B) GSEA enrichment plot for key MSigDB pathways comparing *TP53* altered and *TP53* wild-type groups. (C) GSEA enrichment plot for key MSigDB pathways comparing *NOTCH1* wild-type/*TP53* altered and other groups. (D) Comparison of immune cell infiltration between *NOTCH1* altered and *NOTCH1* wild-type groups. (E) Comparison of immune cell infiltration between *TP53* altered and *TP53* wild-type groups. FDR-adjusted *P*-values are shown for GSEA results in panels A-C. GSEA, gene set enrichment analysis; MSigDB, Molecular Signatures Database.

To further elucidate the underlying mechanisms, we focused on several established clinically relevant pathways in ESCC using the TCGA cohort. The clinicopathologic characteristics of this cohort are presented in Table S1. GSEA results indicated that patients with *NOTCH1* mutations exhibited downregulation of the CYP450 drug metabolism and xenobiotic metabolism pathways (Figure 4A). Conversely, *TP53*-mutated tumors showed significant enrichment of the CYP450, ECM-receptor interaction, and PI3K-Akt signaling pathways (Figure 4B). Moreover, patients with *TP53* mutations, but lacking *NOTCH1* mutations, also exhibited enrichment in CYP450 and chemical carcinogenesis pathways (Figure 4C). All significant GSEA results are detailed in Tables S2-S4.

Finally, immune infiltration analysis revealed that patients without *NOTCH1* alterations exhibited higher monocyte and naive B cell infiltration (Figure 4D), whereas *TP53* mutations correlated with lower levels of memory B cell and resting NK cell infiltration (Figure 4E). No other significant disparities were observed between the groups (Figure S5A and B). Furthermore, no significant difference was noted between the group with *TP53* alterations and wild-type *NOTCH1* compared to other groups (*P* >.05, Figure S5C).

## Discussion

This study characterized mutational landscapes across tumor, surgical margin, and adjacent normal tissues in ESCC in both retrospective and prospective cohorts. We revealed a significant overlap of mutations between tumors and their paired margins, particularly involving *TP53* and *NOTCH1*. Importantly, the presence of shared *TP53* mutations between tumor and margin tissues emerged as a significant prognostic marker for DFS. These results support mutational profiling across tumors and margins as a potential postoperative prognostic tool.

Despite histologically negative margins after R0 resection, molecular residual disease may persist beyond the visible tumor boundary. Our discovery of shared mutations between tumors and tumor-free margins highlights the limitations of conventional pathology and supports the use of NGS-based molecular margin assessment. Although our analysis did not find a statistically significant correlation between the proportion of shared mutations and DFS, patients experiencing recurrence tended to have higher proportions of shared mutations. Critically, all patients with more than 50% shared mutations experienced disease progression. This implies that while the overall genetic overlap may not directly dictate clinical outcomes, specific key genetic factors could be more influential.

Building upon our observation of shared genetic mutations between tumors and margins, we found that a positive *TP53*-MiT status significantly predicted shorter DFS. This finding indicates that residual clonal populations carrying *TP53* mutations could drive recurrence. Moreover, excluding *NOTCH1* mutations from the MiT status strengthened the prognostic association, suggesting that *NOTCH1* mutations may not significantly contribute to recurrence risk. These findings further indicate that only a small subset of mutations is critical for driving tumor recurrence and metastasis, which can substantially impact the clinical outcomes. Identification of such critical mutations is paramount for improving the prognostic assessment. Consequently, MiT status, especially *TP53*-MiT, emerges as a robust biomarker for postoperative risk stratification, potentially guiding decisions on the necessity and intensity of adjuvant therapies.

The pivotal role of *TP53* as a central driver of malignant transformation in ESCC is reinforced by its high mutation frequency in tumor tissues compared to margin and normal tissues, aligning with previous studies.^24^  *TP53*, a well-established tumor suppressor gene, often mutates at a very early stage and accumulates as the disease progresses.^25^^,^^26^ Dysfunction of *TP53* leads to widespread genomic disruption such as chromosomal rearrangements.^27–29^ Furthermore, *TP53* mutations are associated with resistance to chemotherapy and radiotherapy,^25^^,^^30^ and truncating *TP53* mutations serve as independent negative prognostic markers in non-small cell lung cancer.^31^ Conversely, *NOTCH1* mutations were more frequent in margin and normal tissues, which is consistent with prior studies.^32^^,^^33^ Accumulating evidence demonstrates that *NOTCH1*-mutant clones progressively colonize morphologically normal esophageal epithelium as a hallmark of aging.^15^^,^^34^ While wild-type *NOTCH1* is essential for maintaining physiological homeostasis in normal esophageal tissue, its loss-of-function mutations drive the clonal expansion of normal progenitors while paradoxically restraining subsequent tumor growth.^33^ Furthermore, spatial competition among mutant clones in the aging esophagus favors the expansion of highly fit populations with *NOTCH1* mutations within non-neoplastic tissue.^35^^,^^36^ Notably, although *TP53*-mutant clones can also be detected in normal esophageal epithelium, their prevalence is substantially lower than that of *NOTCH1*-mutant clones in aging tissues, whereas *TP53* alterations are highly enriched in established ESCC.^35–37^ Therefore, *NOTCH1* mutations identified in margin tissues may largely reflect age-related clonal mosaicism of normal epithelium rather than residual cancer cell populations with recurrence potential, whereas a positive *TP53*-MiT status represents a far more reliable indicator of true residual tumor cell clones. This biological distinction may explain why *NOTCH1*-MiT was not associated with DFS in our cohort, whereas *TP53*-MiT retained significant prognostic value.

External TCGA analyses further supported the biologic relevance of *TP53* and *NOTCH1* in ESCC. *TP53* mutations were linked to the activation of pathways such as CYP450, ECM-receptor interaction, and PI3K-Akt signaling, which are vital to cancer growth and metastasis.^38^^,^^39^ In contrast, *NOTCH1* mutations were associated with a significant downregulation of these pathways. In addition, tumors with wild-type *TP53* showed higher fractions of immune-reactive cells, such as memory B cells. Conversely, *NOTCH1* wild-type tumors exhibited increased infiltration of immunosuppressive monocytes, which can suppress T cell activity and promote tumor growth.^40^ These findings further confirmed *TP53* alterations could promote tumor progression, while *NOTCH1* alterations may play a less oncogenic role.

However, our study has limitations. The relatively small sample size, particularly within the prospective cohort, may affect the generalizability of our findings and may also have contributed to the statistical instability of the non-*NOTCH1*-MiT survival analysis, as reflected by the extremely wide and unbounded CI in the Cox model. Additionally, we did not assess the potential impact on overall survival or other clinical outcomes. Larger, multicenter studies with extended follow-up and a greater number of outcome events are warranted to validate the independent prognostic value of *TP53*-MiT and to explore the potential for targeted therapies addressing *TP53*-driven recurrence.

## Conclusion

We have introduced the MiT status as a novel and clinically relevant prognostic biomarker in ESCC, particularly focusing on *TP53* mutations. These findings advocate for the incorporation of NGS-based molecular assessments in surgical oncology, moving towards a more personalized approach in ESCC management.

## Supplementary Material

oyag337_Supplementary_Data

## Data Availability

Data sources and handling of the publicly available datasets used in this study are described in the Patients and Methods section. Further details and other data that support the findings of this study are available from the corresponding authors upon request.
