# Supplementary material for: Prognostic significance of positive margin-informed tumor status in esophageal squamous cell carcinoma using next-generation sequencing
Source: Oncologist. 2026 Aug 22;31(9):oyag337. doi: 10.1093/oncolo/oyag337 (PMC13544954; doi:10.1093/oncolo/oyag337)
Supplement: Supplementary file 1 [file oyag337_supplementary_data.zip › Supplementary Figures.docx]

**Supplementary Figures**

[Supplementary Figure S1. Mutation information and patterns of shared mutations between tumor and margin tissues in the retrospective cohort.	2](#_Toc233293715)

[Supplementary Figure S2. Mutation frequencies and mutation count trends of *NOTCH1* and *TP53* in the retrospective and prospective cohorts.	4](#_Toc233293716)

[Supplementary Figure S3. Mutation information and patterns of shared mutations between tumor and margin tissues in the prospective cohort.	5](#_Toc233293717)

[Supplementary Figure S4. Associations between margin status and DFS, MiT statuses clinicopathological parameters, and independent prognostic values of MiT in retrospective and prospective cohorts.	7](#_Toc233293718)

[Supplementary Figure S5. Detailed immune infiltration analysis based on CIBERSORT algorithm.	9](#_Toc233293719)

# Supplementary Figure S1. Mutation information and patterns of shared mutations between tumor and margin tissues in the retrospective cohort.

**(A)** The distribution of somatic mutation counts across 48 tumor samples, 48 margin samples and 33 normal samples. Each raincloud plot combines a half-violin showing density distribution, a boxplot displaying the median (horizontal line), interquartile range (IQR, box boundaries representing 25th to 75th percentiles), and individual data points with horizontal jittering to avoid overlapping of identical values. Red triangles indicate mean values for each tissue type. Numerical values for both mean and median mutation counts are displayed above each group for precise reference. Different colors distinguish each tissue type category.

**(B)** Network of shared mutations among tissue types. The diagram displays a network graph depicting the proportion of patients carrying shared mutations in paired tissues. Gradient color schemes are used, with more intense red colors indicating a higher proportion of patients with shared mutations between the tissue types.

**(C)** Pie plot of shared and non-shared mutations of the eight patients.

**(D)** Bar plot of the frequency of shared mutated genes between tumor and margin tissues. Each bar represents a gene, with bar height and color intensity (red) proportional to the frequency of shared mutations between tumor and margin tissues. Genes with higher mutation frequencies are depicted by taller bars with deeper red coloration.

**(E)** The proportion of shared and non-shared mutations per patient. Bar charts illustrating the percentage of mutations classified as shared (present in both tumor and margin tissues), tumor-only (exclusive to tumor tissue), and margin-only (exclusive to margin tissue) for each of the eight patients exhibiting shared mutations in the retrospective cohort. The proportion of shared mutations varies among patients, ranging from 6.7% to 83.3%.

**(F)** Relationship between shared mutations and total mutations per patient. Bubble plot showing the proportion of shared mutations relative to the total number of mutations for each patient, categorized by DFS status (disease progression vs. disease-free). Each bubble represents a patient, with the *x*-axis indicating the total mutation count and the *y*-axis indicating the shared mutation count. A gray dashed line marks the 50% shared mutation proportion. Patients with a shared mutation proportion exceeding 50% (P02 and P04) are highlighted, both corresponding to cases with disease progression.

**(G)** Comparison of shared mutation proportions by disease progression status. Boxplot comparing the proportion of shared mutations between patients who experienced disease progression and those who remained disease-free. Statistical significance was assessed using the Wilcoxon rank-sum test.

IQR, interquartile range; ns, not significant; DFS, disease-free progression.

# Supplementary Figure S2. Mutation frequencies and mutation count trends of *NOTCH1* and *TP53* in the retrospective and prospective cohorts.

**(A)** Fractions of patients with *NOTCH1* and *TP53* mutations across tumor, margin and normal tissues in the retrospective cohort.

**(B)** Jonckheere-Terpstra test and median mutation counts for *NOTCH1* and *TP53* across tumor, margin, and normal tissues in the retrospective cohort.

**(C)** Fractions of patients with *NOTCH1* and *TP53* mutations across tumor and margin tissues in the prospective cohort.

**(D)** Jonckheere-Terpstra test and median mutation counts for *NOTCH1* and *TP53* across tumor and margin tissues in the prospective cohort.

JT test, Jonckheere-Terpstra test.

# Supplementary Figure S3. Mutation information and patterns of shared mutations between tumor and margin tissues in the prospective cohort.

**(A)** Mutation profiles and clinical features of samples in the prospective cohort. The mutation profiles and clinical features of 22 tumor samples and 22 margin samples with detectable mutations of the prospective cohort were displayed in the left and right panels, respectively. Only the top 25 most frequently altered genes in the cohort were included. Each column represents an individual patient within the cohort. The upper sections illustrate clinicopathologic subtypes of patients, while the bottom sections depict different mutation types of genomic alterations.

**(B)** The distribution of somatic mutation counts across 22 tumor samples and 22 margin samples. Each raincloud plot combines a half-violin showing density distribution, a boxplot displaying the median (horizontal line), interquartile range (IQR, box boundaries representing 25th to 75th percentiles), and individual data points with horizontal jittering to avoid overlapping of identical values. Red triangles indicate mean values for each tissue type. Numerical values for both mean and median mutation counts are displayed above each group for precise reference. Different colors distinguish each tissue type category.

**(C)** Frequency of shared and unique gene alterations between tumor and margin tissues.

**(D)** Pie plot of shared and non-shared mutations of the ten patients.

**(E)** Bar plot of the frequency of shared mutated genes between tumor and margin tissues. Each bar represents a gene, with bar height and color intensity (red) proportional to the frequency of shared mutations between tumor and margin tissues. Genes with higher mutation frequencies are depicted by taller bars with deeper red coloration.

**(F)** The proportion of shared and non-shared mutations per patient. Bar charts illustrating the percentage of mutations classified as shared (present in both tumor and margin tissues), tumor-only (exclusive to tumor tissue), and margin-only (exclusive to margin tissue) for each of the ten patients exhibiting shared mutations in the prospective cohort. The proportion of shared mutations varies among patients, ranging from 4.0% to 85.7%.

**(G)** Relationship between shared mutations and total mutations per patient. Bubble plot showing the proportion of shared mutations relative to the total number of mutations for each patient, categorized by DFS status (disease progression vs. disease-free). Each bubble represents a patient, with the *x*-axis indicating the total mutation count and the *y*-axis indicating the shared mutation count. A gray dashed line marks the 50% shared mutation proportion. Patients with a shared mutation proportion exceeding 50% (P11, P14 and P17) are highlighted, both corresponding to cases with disease progression.

**(H)** Comparison of shared mutation proportions by disease progression status. Boxplot comparing the proportion of shared mutations between patients who experienced disease progression and those who remained disease-free. Statistical significance was assessed using the Wilcoxon rank-sum test.

IQR, interquartile range; ns, not significant; DFS, disease-free progression.

# Supplementary Figure S4. Associations between margin status and DFS, MiT statuses clinicopathological parameters, and independent prognostic values of MiT in retrospective and prospective cohorts.

**(A)** Kaplan‒Meier curves of DFS comparing positive margin status and negative margin status groups in the retrospective cohort.

**(B)** Kaplan‒Meier curves of DFS comparing positive margin status and negative margin status groups in the prospective cohort.

**(C)** Comparison of clinicopathological parameters between different *TP53*-MiT statuses in the retrospective cohort.

**(D)** Comparison of clinicopathological parameters between different *TP53*-MiT statuses in the prospective cohort.

**(E)** Comparison of clinicopathological parameters between different non-*NOTCH1*-MiT statuses in the retrospective cohort.

**(F)** Comparison of clinicopathological parameters between different non-*NOTCH1*-MiT statuses in the prospective cohort.

The left panel displays the mutation frequency distributions of the clinicopathological parameters between MiT-positive and MiT-negative groups. The numbers and percentages indicate the proportion of patients with a particular clinicopathological feature in each group. The right panel shows the corresponding forest plots with odds ratios (ORs) and 95% confidence intervals (CIs) for the association between MiT status and each clinicopathological parameter. ORs are represented with diamonds, and 95% CIs are shown, with truncated intervals indicated by arrows.

MiT, margin-informed tumor; DFS, disease-free survival; Neg, negative; Pos, positive; LNM, lymph node metastasis; OR, odds ratio; CI, confidence interval.

# Supplementary Figure S5. Detailed immune infiltration analysis based on CIBERSORT algorithm.

Box plots illustrating the differential infiltration of immune cell types between *NOTCH1*-mutated (*NOTCH1*+) and wild-type (*NOTCH1*-) groups **(A)**, between *TP53*-mutated (*TP53+*) and wild-type (*TP53-*) samples **(B)**, and based on the *TP53* and *NOTCH1* mutation status (*TP53*+*NOTCH1*- vs. all other samples) **(C)**. The proportions of 22 immune cell types were estimated using the CIBERSORT algorithm. Statistical significance of differences between groups was determined using the Wilcoxon rank-sum test, with *p*-values indicating significant variations in specific immune cell populations between groups. **p* < 0.05, ***p* < 0.01.
